# Albumin treatment regimen for type 1 hepatorenal syndrome: a dose–response meta-analysis

**DOI:** 10.1186/s12876-015-0389-9

**Published:** 2015-11-25

**Authors:** Francesco Salerno, Roberta J. Navickis, Mahlon M. Wilkes

**Affiliations:** 1 Dipartimento di Medicina Interna, Università degli Studi di Milano, Policlinico IRCCS San Donato, Via Morandi 30, 20097 Milano, Italy; 2 Hygeia Associates, 17988 Brewer Rd., Grass Valley, California 95949 USA

**Keywords:** Type 1 hepatorenal syndrome, Albumin, Mortality, Dose–response relationship, drug

## Abstract

**Background:**

Recommended treatment for type 1 hepatorenal syndrome consists of albumin and vasoconstrictor. The optimal albumin dose remains poorly characterized. This meta-analysis aimed to determine the impact of albumin dose on treatment outcomes.

**Methods:**

Clinical studies of type 1 hepatorenal syndrome treatment with albumin and vasoconstrictor were sought. Search terms included: hepatorenal syndrome; albumin; vasoconstrictor; terlipressin; midodrine; octreotide; noradrenaline; and norepinephrine. A meta-analysis was performed of hepatorenal syndrome reversal and survival in relation to albumin dose.

**Results:**

Nineteen clinical studies with 574 total patients were included, comprising 8 randomized controlled trials, 8 prospective studies and 3 retrospective studies. The pooled percentage of patients achieving hepatorenal syndrome reversal was 49.5 % (95 % confidence interval, 40.0-59.1 %). Increments of 100 g in cumulative albumin dose were accompanied by significantly increased survival (hazard ratio, 1.15; 95 % confidence interval, 1.02-1.31; p = 0.023). A non-significant increase of similar magnitude in hepatorenal syndrome reversal was also observed (odds ratio, 1.15; 95 % confidence interval, 0.97-1.37; p = 0.10). Expected survival rates at 30 days among patients receiving cumulative albumin doses of 200, 400 and 600 g were 43.2 % (95 % confidence interval, 36.4-51.3 %), 51.4 % (95 % confidence interval, 46.3-57.1 %) and 59.0 % (95 % confidence interval, 51.9-67.2), respectively. Neither survival nor hepatorenal syndrome reversal was significantly affected by vasoconstrictor dose or type, treatment duration, age, baseline serum creatinine, bilirubin or albumin, baseline mean arterial pressure, or study design, size or time period.

**Conclusions:**

This meta-analysis suggests a dose–response relationship between infused albumin and survival in patients with type 1 hepatorenal syndrome. The meta-analysis provides the best current evidence on the potential role of albumin dose selection in improving outcomes of treatment for type 1 HRS and furnishes guidance for the design of future dose-ranging studies.

**Electronic supplementary material:**

The online version of this article (doi:10.1186/s12876-015-0389-9) contains supplementary material, which is available to authorized users.

## Background

Hepatorenal syndrome (HRS) is a form of functional severe renal failure in patients with advanced liver cirrhosis. This life-threatening complication results from marked renal vasoconstriction and its consequent reduced renal perfusion and glomerular filtration rate. The diagnosis is based mainly on excluding other causes of renal impairment and demonstrating lack of response to a 2-day course of volume expansion [1]. Based on clinical features and prognosis, HRS is classified into type 1 and 2. Type 1 HRS presents as acute renal failure characterized by at least a two-fold increase in serum creatinine to a level greater than 2.5 mg/dL in less than 2 weeks. The prognosis is poor, with survival averaging 11 days in patients with untreated type 1 HRS [2]. However, this condition is potentially reversible, and renal function and survival can be improved by prompt medical treatment in about half of the patients developing type 1 HRS.

As a first-line therapy of type 1 HRS, both the International Ascites Club (IAC) and the American Association for the Study of Liver Diseases (AASLD) recommend a combination of vasoconstrictors and albumin infusion [3, 4]. The additive effects provided by vasoconstrictors and albumin infusion are thought to improve outcomes vs. monotherapy with either agent. The vasopressors shown in clinical studies to be effective have been primarily either terlipressin, a vasopressin analogue, or the α-agonist midodrine combined with octreotide [3, 4]. Noradrenaline is another option. Patients are typically titrated with escalating vasoconstrictor doses until a response to treatment is achieved [3, 4].

Albumin has generally been administered in fixed doses. The IAC guidelines [3] recommend an albumin dose of 1 g/kg on the first day up to a maximum of 100 g followed by 20–40 g/day. Nevertheless, dose optimization studies have not been reported, and questions remain about the most effective albumin regimen. Meta-analysis provides a long established and commonly applied methodology for quantitatively combining dose–response data across studies [5, 6]. This meta-analysis was designed to evaluate the impact of albumin dose selection on outcomes of type 1 HRS.

## Patients and methods

### Study selection

Clinical studies were sought which evaluated albumin infusion and concomitant vasoconstrictor to treat patients with type 1 HRS. Case reports and case series were excluded. Data must have been available on HRS reversal and/or survival time. Albumin and vasoconstrictor dose and duration of treatment must have been reported. Studies of ornipressin, vasopressin, monotherapy with octreotide, and furosemide co-therapy were not considered. No restrictions were placed on study design, prior publication, language of reporting or time period. Study eligibility was determined by all three investigators.

While randomized trials were sought, in no case were type 1 HRS patients randomly assigned to different albumin doses. Thus, although random allocation might signify higher study quality, it could not serve to minimize potential confounding in this meta-analysis. Nonrandomized studies were also sought. Inclusion of such studies in meta-analyses, where feasible, has been advocated because they can increase statistical power as well as allowing important clinical questions to be addressed for which randomized trial data are unavailable or inadequate [7, 8]. Nonrandomized studies may be more vulnerable to biases, but nonetheless empirical studies have shown treatment effects to be similar between randomized and nonrandomized studies [9, 10]. Based upon a representative cross-sectional sample of 300 published meta-analyses, nonrandomized studies were eligible for inclusion in 40 % of all meta-analyses [11].

### Search strategy

Sources searched for eligible studies were MEDLINE, EMBASE, the Cochrane Library, the ClinicalTrials.gov web site, the abstract databases from major meetings in hepatology and gastroenterology, texts indexed by Google, reference lists of publications on HRS, and online tables of contents for hepatology and gastroenterology journals. Computer search terms included hepatorenal syndrome; albumin; vasoconstrictor; terlipressin; midodrine; octreotide; noradrenaline; and norepinephrine; as well as roots and variants of those terms. A representative MEDLINE search strategy is outlined in Table 1.

**Table 1:** Representative MEDLINE search strategy

Set	Query
1	“hepatorenal syndrome”
2	albumins/therapeutic use [mh]
3	vasoconstrictor* OR terlipressin OR midodrine OR octreotide OR noradrenaline OR norepinephrine
4	lypressin/therapeutic use [mh]
5	adrenergic alpha-agonists/therapeutic use [mh]
6	#3 OR #4 OR #5
7	humans [mh]
8	#1 AND #2 AND #6 AND #7

### Data extraction

Details of candidate study reports were scrutinized to avoid inclusion of redundant data appearing in multiple articles. Data were extracted for year of reporting; study design; criteria for diagnosis of HRS and for HRS reversal; numbers of patients; age; baseline serum creatinine, bilirubin and albumin; creatinine increase after diagnostic volume expansion; frequencies of ascites, spontaneous bacterial peritonitis, infection, gastrointestinal bleeding and hepatocellular carcinoma; baseline mean arterial pressure, heart rate, hemoglobin, white blood cells, and platelets; albumin and vasoconstrictor dose; concentration of infused albumin; duration of treatment; HRS reversal; adverse events due to albumin administration and survival time. In controlled investigations data were extracted only for study arms fulfilling the eligibility criteria of the meta-analysis. When data for patients with either type 1 or 2 HRS were presented in segregated form, only the type 1 data were extracted. Type 1/2 HRS outcome data reported only in the aggregate were excluded. Individual patient serum creatinine and survival time data were captured as required by computer digitization from graphic displays in the study reports. Data extraction was carried out independently by two investigators, and differences of interpretation were discussed and resolved. When necessary, included study investigators were contacted for supplementary unpublished data.

### Ethical approval and consent

This investigation was limited to statistical analysis of data collected in previous clinical studies and did not constitute medical research involving human subjects or research on identifiable human material and data. Accordingly, ethics committee approval and patient informed consent were not applicable.

### Standards of reporting

This meta-analysis was reported in conformity with the Meta-analysis Of Observational Studies in Epidemiology (MOOSE) guidelines [7]. A completed MOOSE checklist is included in Additional file 1.

### Data availability

The data sets supporting the results of this meta-analysis are provided in Additional file 2 as working R code (The R Foundation for Statistical Computing, Vienna, Austria). Raw data, function definitions and intermediate computations are included.

### Statistical analysis

The hypothesis of the meta-analysis was that outcomes of type 1 HRS treatment vary significantly as a function of albumin dose. The endpoints were HRS reversal, defined as reduction in serum creatinine to < 1.5 mg/dL or ¶ 1.5 mg/dL, and survival. If other HRS reversal criteria were applied in the reports of included studies, the individual patient serum creatinine data were used to reclassify the patients in accordance with the < 1.5 mg/dL criterion of the meta-analysis.

It was anticipated that variation in albumin doses administered would result in significant heterogeneity of outcomes between patient groups. Heterogeneity was assessed by Cochran Q test and the I^2^ statistic [12]. In order to accommodate expected heterogeneity, random effects models were used to combine results quantitatively across studies. Albumin dose was analyzed as one source of heterogeneity. Also, in accordance with the MOOSE guidelines [7], an array of other variables were examined as potential sources of heterogeneity.

Since patients with type 1 HRS are subject to rapidly increasing mortality, crude mortality data are vulnerable to bias resulting from differences in duration of follow-up, and only survival data with accounting for time at risk were used in the meta-analysis. In the analysis of survival, patients were censored at the time of loss to follow-up or supervening interventions such as liver transplantation or transjugular intrahepatic portosystemic shunt insertion. Individual study group survival curves were analyzed by the Kaplan-Meier product-limit method. The effects of albumin dose and other variables on HRS reversal and survival were analyzed by mixed effects logistic regression and Cox proportional hazards regression, respectively, with study as a random effect in both cases. Study quality and publication bias were assessed by sensitivity analyses of study design and size, respectively. Statistical analyses were conducted using R version 3.0.2 software.

## Results

### Included studies

**Fig. 1:**
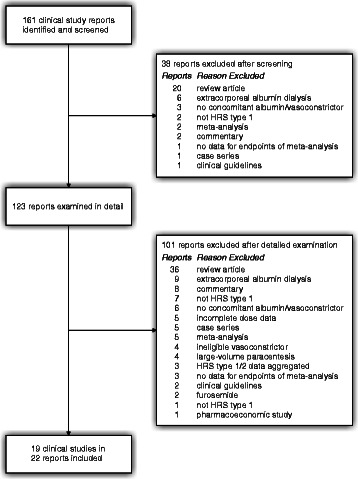
Clinical study selection process. Abbreviation: HRS, hepatorenal syndrome

The selection process for the meta-analysis is shown in Fig. 1. Of 161 candidate clinical study reports identified, 38 were excluded at the screening stage, most often because they were review articles. After detailed examination of the remaining 123 reports, 101 were excluded, again most frequently since they reviewed the literature rather than providing original data.

Upon determination that they fulfilled all eligibility criteria 19 clinical studies published from 1999 to 2012 with 574 total patients were included in the meta-analysis [1, 13–30]. The median number of patients per study was 24 (interquartile range, 12–44). Data from 4 of the studies [14, 16, 19, 21] were also included in 3 separate publications focused on predictors of response to treatment [31–33]. Eight of the 19 included studies were randomized controlled trials, 8 prospective studies and 3 retrospective studies (Tables 2 and 3). Two arms of 4 randomized trials and of one prospective study were included, and thus 24 total patient groups were represented in the meta-analysis (Tables 2 and 3).

**Table 2:** Treatment regimen^a^

Study	Patients	Albumin dose (g)	Vasoconstrictor		Treatment duration (d)
Randomized			*Type*	*Dose (mg)*	
Alessandria et al., 2007 [18]	5	350	terlipressin	55	7.6
	4	406	noradrenaline	188	7.2
Martín-Llahí et al., 2008 [19]	17	190	terlipressin	66	7.0
Neri et al., 2008 [20]	26	836	terlipressin	36	19.0
Sanyal et al., 2008 [21]	56	304	terlipressin	28	6.3
Sharma et al., 2008 [22]	20	243	terlipressin	47	8.1
	20	234	noradrenaline	187	7.8
Silawat et al., 2011 [27]	30	88	terlipressin	10	7.0
Singh et al., 2012 [29]	23	156	terlipressin	24	7.8
	23	186	noradrenaline	132	9.3
Tavakkoli et al., 2012 [30]	9	720	midodrine/octreotide	270/5.4	18.0
	6	720	noradrenaline	146	18.0
Prospective					
Angeli et al., 1999 [13]	5	300	midodrine/octreotide	630/9.6	20.0
Uriz et al., 2000 [14]	6	358	terlipressin	48	10.6
Mulkay et al., 2001 [15]	12	200	terlipressin	72	26.0
Wong et al., 2004 [17]	14	700	midodrine/octreotide	35/8.4	14.0
Muñoz et al., 2009 [23]	13	592	terlipressin	41	9.6
Rivero et al., 2010 [26]	41	250	terlipressin	63	7.0
Salerno et al., 2011 [1]	40	235	terlipressin	63	8.7
	24	235	midodrine/octreotide	207/2.8	8.7
Narahara et al., 2012 [28]	8	162	terlipressin	18	6.3
Moreau et al., 2002 [16]	99	433	terlipressin	36	11.4
Skagen et al., 2009 [24]	49	368	midodrine/octreotide	222/3.6	8.4
von Kalckreuth et al., 2009 [25]	24	193	terlipressin	27	7.1

^a^Indicated doses are mean cumulative values.

**Table 3:** Baseline patient characteristics^a^

Study	Vasoconstrictor	Age (y)	Serum concentration^b^			MAP (mm Hg)
Randomized			*Creatinine*	*Bilirubin*	*Albumin*	
Alessandria et al., 2007 [18]^c^	terlipressin	55.0 (6.9)	2.5 (1.0)	5.1 (3.5)	3.0 (0.3)	74.0 (10.4)
	noradrenaline	56.0 (9.5)	2.3 (0.6)	4.1 (3.2)	3.0 (0.6)	71.0 (6.3)
Martín-Llahí et al., 2008 [19]^c^	terlipressin	59.0 (10.0)	3.6 (1.5)	18.1 (19.1)	3.0 (0.7)	73.0 (10.0)
Neri et al., 2008 [20]	terlipressin	59.0 (4.0)	2.8 (1.1)	—	2.7 (0.3)	82.0 (2.0)
Sanyal et al., 2008 [21]	terlipressin	50.6 (10.5)	4.0 (2.2)	15.0 (13.6)	2.6 (0.8)	75.5 (11.4)
Sharma et al., 2008 [22]	terlipressin	47.8 (9.8)	3.0 (0.5)	7.6 (9.8)	2.6 (0.6)	81.4 (11.4)
	noradrenaline	48.2 (13.4)	3.3 (1.3)	5.2 (6.8)	2.4 (0.4)	78.2 (5.3)
Silawat et al., 2011 [27]	terlipressin	—	3.0 (1.3)	3.4 (2.1)	2.4 (0.7)	67.6 (16.4)
Singh et al., 2012 [29]	terlipressin	51.4 (11.6)	3.3 (0.7)	4.0 (2.6)	2.8 (0.4)	64.7 (11.9)
	noradrenaline	48.3 (11.6)	3.1 (0.7)	4.7 (5.7)	2.8 (0.2)	65.2 (10.2)
Tavakkoli et al., 2012 [30]^c^	midodrine/octreotide	52.9 (12.6)	2.6 (0.8)	11.6 (12.2)	2.6 (0.3)	69.8 (7.1)
	noradrenaline	52.0 (12.9)	2.6 (0.7)	8.0 (7.8)	2.7 (0.2)	73.4 (6.7)
Prospective						
Angeli et al., 1999 [13]	midodrine/octreotide	62.0 (6.7)	5.0 (2.0)	4.3 (2.9)	3.0 (0.2)	75.9 (6.7)
Uriz et al., 2000 [14]^c^	terlipressin	54.0 (11.0)	3.9 (2.1)	14.0 (18.0)	3.2 (0.6)	68.0 (6.0)
Mulkay et al., 2001 [15]	terlipressin	53.5 (5.1)	3.4 (0.5)	6.2 (6.3)	2.9 (0.3)	76.0 (5.0)
Wong et al., 2004 [17]	midodrine/octreotide	55.2 (7.9)	2.9 (1.2)	3.0 (1.6)	3.2 (1.0)	80.6 (14.7)
Muñoz et al., 2009 [23]	terlipressin	54.2 (21.5)	3.3 (5.9)	—	—	69.6 (29.6)
Rivero et al., 2010 [26]	terlipressin	—	—	—	—	—
Salerno et al., 2011 [1]	terlipressin	62.0 (7.6)	3.2 (1.2)	15.1 (10.1)	2.8 (0.4)	80.4 (8.2)
	midodrine/octreotide	62.0 (5.9)	3.2 (0.9)	15.1 (7.8)	2.8 (0.3)	80.4 (6.4)
Narahara et al., 2012 [28]	terlipressin	59.1 (11.8)	3.0 (0.8)	9.4 (7.6)	2.5 (0.4)	74.0 (14.0)
Retrospective						
Moreau et al., 2002 [16]	terlipressin	56.0 (10.0)	2.9 (1.1)	11.8 (12.9)	2.8 (0.7)	78.0 (20.0)
Skagen et al., 2009 [24]	midodrine/octreotide	52.7 (10.6)	2.5 (1.3)	—	—	—
von Kalckreuth et al., 2009 [25]	terlipressin	51.8 (9.4)	—	—	—	—

^a^Indicated values are mean (standard deviation).

^b^Serum creatinine and serum bilirubin in mg/dL and serum albumin in g/dL.

^c^Indicated baseline data reported only in the aggregate for types 1 and 2 HRS. However, outcome data (HRS reversal/survival) were reported separately for patients with type 1HRS, and only those separate outcome data for type 1 HRS were used in the meta-analysis.

Abbreviation: HRS, hepatorenal syndrome; MAP, mean arterial pressure.

In all 19 studies type 1 HRS was diagnosed according to the IAC criteria [3, 34]. Diagnostic volume expansion was with albumin in 6 studies [1, 17, 18, 22, 23, 30], saline in 3 [20, 25, 28], both in 2 [13, 15] and either or both in one [21]. Unspecified colloid or crystalloid was utilized for diagnostic volume expansion in one study [24], while type of fluid infused for that purpose was unspecified in 5 studies [14, 16, 19, 27, 29].

### Treatment

The mean cumulative dose of albumin administered was < 200 g in 7 of the patient groups (29.2 %), 200–400 g in 10 (41.6 %) and > 400 g in 7 (29.2 %), as shown in Table 2. The daily albumin dose averaged < 30 g in 10 groups (41.6 %), 30–40 g in 7 (29.2 %) and > 40 g in 7 (29.2 %). The concentration of albumin infused was 20 % in all 10 groups for which specified.

Albumin was co-administered with terlipressin in 15 groups (62.5 %), midodrine/octreotide in 5 (20.8 %) and noradrenaline in 4 (16.7 %). The mean duration of albumin/vasoconstrictor therapy was < 8 d in 10 groups (41.7 %), 8–14 d in 9 (37.5 %) and > 14 d in 5 (20.8 %).

### Patients

Five baseline patient characteristics were consistently reported for the study groups: age, serum concentrations of creatinine, bilirubin and albumin, and mean arterial pressure (Table 3). Other baseline variables were more sparsely reported. Data were presented on the baseline frequency of infection for 16 groups, ascites for 13, gastrointestinal bleeding for 9, hepatocellular carcinoma for 8, and spontaneous bacterial peritonitis for 2. Patients exhibiting an increase in serum creatinine after diagnostic volume expansion were enumerated for one group. Measurements were reported of heart rate for 11 groups, hemoglobin and platelets for 3 each and white blood cells for 2.

In 7 reports [16, 17, 22, 23, 29, 32, 33] baseline patient data were stratified according to treatment response. Significant differences for the 5 variables in Table 3 were infrequent and inconsistent. Mean arterial pressure was significantly higher among responders than nonresponders in 2 reports [22, 29] as also was serum albumin in one of those 2 reports [29]. Responders were significantly younger than nonresponders in one report [16], while baseline bilirubin was lower among responders in another report [32].

### HRS reversal

In 15 studies the criterion for HRS reversal was a decline in serum creatinine to < 1.5 mg/dL or the reported data allowed classification of the patients in accordance with that cutoff. In 3 studies the criterion was ¶ 1.5 mg/dL. No criterion was indicated for one study assessing renal function and survival but not HRS reversal *per se* [24].

**Fig. 2:**
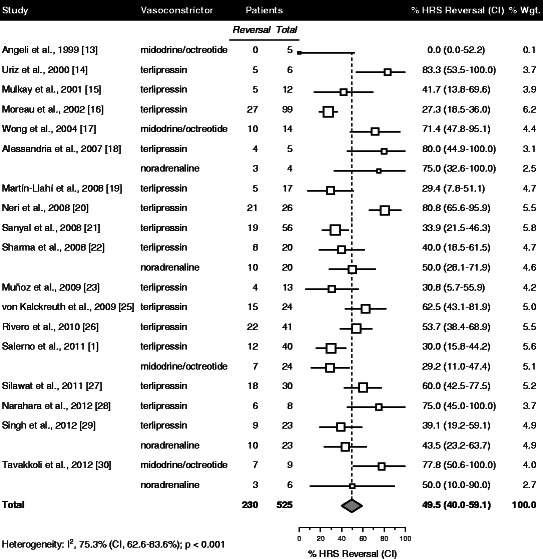
HRS reversal. Data points scaled in proportion to meta-analytic weight under a random effects model. Error bars represent CI. Abbreviations: CI, 95 % confidence interval; HRS, hepatorenal syndrome

Data on HRS reversal were available for 23 patient groups with 525 total patients (Table 4). The pooled percentage of patients attaining HRS reversal was 49.5 % with a 95 % confidence interval (CI) of 40.0-59.1 % (Fig. 2). No significant differences in HRS reversal could be detected in relation to any of the treatment, patient and study variables listed in Table 4.

**Table 4:** Effect of treatment, patient and study variables on outcome

Variable^a^	HRS Reversal			Survival		
Treatment	*Groups*	*Odds Ratio (CI)*	*p*	*Groups*	*Hazard Ratio (CI)*	*p*
Albumin dose (g)	23	1.15 (0.97-1.37)	0.10	15	1.15 (1.02-1.31)	0.023
Vasoconstrictor dose (mg)	23	0.86 (0.61-1.20)	0.38	15	1.28 (0.91-1.79)	0.16
Vasoconstrictor type	23	0.90 (0.51-1.57)	0.70	15	0.89 (0.49-1.59)	0.69
Treatment duration (d)	23	1.15 (0.46-2.89)	0.76	15	1.63 (0.84-3.13)	0.15
Patient						
Age (y)	21	0.99 (0.39-2.51)	0.99	15	1.37 (0.78-2.42)	0.28
Serum creatinine (mg/dL)	21	0.56 (0.31-1.01)	0.054	15	0.93 (0.52-1.65)	0.79
Serum bilirubin (mg/dL)	19	0.76 (0.37-1.57)	0.46	12	1.13 (0.65-1.94)	0.67
Serum albumin (g/dL)	20	1.11 (0.41-2.99)	0.83	13	1.58 (0.80-3.12)	0.19
MAP (mm Hg)	21	1.02 (0.41-2.55)	0.97	14	1.24 (0.71-2.15)	0.45
Study						
Design						
Prospective	11	1.24 (0.36-4.29)	0.74	7	1.05 (0.55-1.99)	0.88
Randomized	21	1.25 (0.56-2.84)	0.59	13	0.85 (0.44-1.64)	0.62
Number of patients	23	0.68 (0.45-1.04)	0.07	15	0.95 (0.68-1.33)	0.75
Year reported	23	1.38 (0.56-3.40)	0.48	15	0.84 (0.44-1.59)	0.58

^a^Albumin dose was analyzed per 100 g increment in cumulative dose. To allow comparisons between doses of different vasoconstrictors on a common scale, cumulative doses of each vasoconstrictor were standardized, with the values for midodrine and octreotide averaged. In the analysis of vasoconstrictor type, terlipressin was compared with other vasoconstrictors. To normalize data distributions, treatment duration and number of patients were log transformed. Patient variables were stratified by values above cutoffs equaling the pooled midpoint between the means of responders and nonresponders among the included studies as compared with lower values. The cutoffs were 53 y for age, 3.1 mg/dL for serum creatinine, 8.3 mg/dL for serum bilirubin, 2.8 g/dL for serum albumin, and 76 mm Hg for MAP. Prospective studies were compared with retrospective studies as the reference category, randomized studies with nonrandomized studies, and studies reported after 2005 with those before. Available data for HRS reversal were from 23 patient groups with 525 total patients and for time of survival from 15 patient groups with 377 total patients.

Abbreviations: HRS, hepatorenal syndrome; MAP, mean arterial pressure.

### Survival

**Fig. 3:**
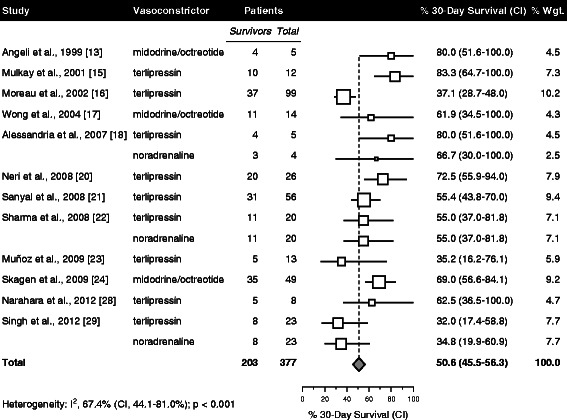
Survival at 30 days. Graphic conventions as in Fig. 2. Abbreviation: CI, 95 % confidence interval

Time of survival data were reported for 15 patient groups with 377 total patients (Fig. 3). At 30 days, pooled survival was 50.6 % (CI, 45.5-56.3 %).

Increments of 100 g in cumulative albumin dose were associated with a significant increase in survival (hazard ratio, 1.15; CI, 1.02-1.31; p = 0.023; Table 4). An association of similar magnitude and direction was also observed for 10 g increments in mean daily albumin dose; however, that association did not reach statistical significance (hazard ratio, 1.20; CI, 0.99-1.46); p = 0.064). No significant survival differences were observed with respect to any of the other tested variables (Table 4).

**Fig. 4:**
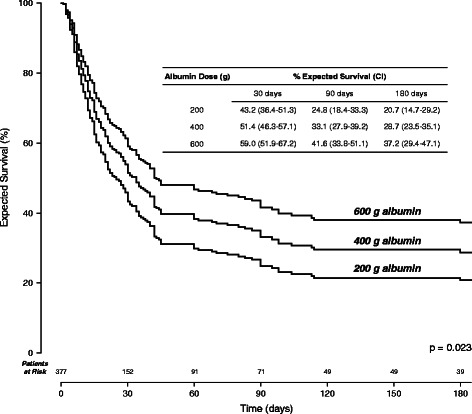
Expected survival in patients receiving cumulative albumin doses of 200, 400 and 600 g. Abbreviation: CI, 95 % confidence interval

From the Cox regression model expected survival curves were constructed for several cumulative albumin doses (Fig. 4). Among patients receiving 200, 400 and 600 g cumulative doses the expected percentages surviving at 30 days were 43.2, 51.4 and 59.0 %, respectively.

## Discussion

A number of previous meta-analyses have examined pharmacologic treatment of HRS [35–39]. However, they all focused on the effectiveness of vasoconstrictor treatment, and indeed only one even required concomitant albumin as an inclusion criterion [38]. The present meta-analysis is the first to investigate the impact of albumin dose on treatment outcomes of type 1 HRS.

The outlook for type 1 HRS patients has improved markedly with the advent of pharmacological therapy employing vasoconstrictors and albumin in the last 15 years. This approach has provided for some patients a successful bridge to liver transplantation, the only definitive treatment for HRS [3, 40–42]. The goal is to reverse in a very short time window the kidney failure before it leads to irreversible structural renal damage and death. However, only an estimated 40 to 60 % of patients respond to the combination therapy with reversal of kidney failure [43, 44]. Consequently, ways to improve the efficacy of the combined treatment and to identify predictors of response are currently active areas of investigation [31–33].

While the primary focus for optimizing therapy has been on the dose, duration and mode of administration for the different vasoconstrictors [45, 46], albumin is recognized as an integral part of the therapy and optimization of the albumin regimen could also potentially lead to improved response and survival. While the regimen of albumin administered has not been the primary endpoint of any individual study, the numerous currently available reports on the use of vasoconstrictors and albumin in type 1 HRS patients allow the examination of treatment outcomes as a function of the albumin regimen over a substantial range of cumulative albumin doses.

Across all available studies included in the meta-analysis the overall rate of HRS reversal in response to co-therapy with albumin and vasoconstrictor was 49.5 %. This is the most comprehensive and precise estimate of HRS reversal rate thus far and confirms the accuracy of prior estimates. Importantly, while a variety of definitions for HRS reversal have been adopted in individual studies, the estimated rate in the meta-analysis was based upon a single homogeneous criterion of serum creatinine reduction to < 1.5 mg/dL or ¶ 1.5 mg/dL. This strength of the meta-analysis was made possible by extraction and use of individual patient serum creatinine data.

While 100 g increments in cumulative albumin dose were associated with increased HRS reversal rate (Table 4), this association did not reach statistical significance (p = 0.10). Nor did any other evaluated treatment, patient or study variable show a significant effect on HRS reversal. Some differences in baseline patient variables related to treatment response were observed in individual studies, although these were infrequent and inconsistent.

The meta-analysis revealed significant improvement in survival associated with 100 g increments in cumulative albumin dose (Table 4). Over the range of cumulative albumin doses between 200 and 600 g, a range commonly administered among the included studies, expected survival at 30 days increased from 43.2 to 59.0 % (Fig. 4). The robustness of these findings is supported by the relatively large sample size of 377 patients at risk for death; the extraction of individual patient survival time data and analysis of those data by methodology that fully accounts for time at risk; and the ability to rule out confounding by an array of treatment, patient and study variables. The association between increasing cumulative albumin dose and decreasing mortality could not be explained by vasoconstrictor dose or type, treatment duration, age, baseline serum creatinine, bilirubin or albumin, baseline mean arterial pressure, or study design, size or time period. On the other hand, the investigations included in the meta-analysis were not specifically designed as dose-ranging studies with head-to-head dose comparisons, and there remains the possibility of confounding by variables reported either only infrequently or not at all.

Furthermore, association does not imply causation. Patients surviving longer might simply be receiving higher cumulative albumin doses as a result. If so, it could be expected that longer survival would be associated with a more extended duration of treatment. However, that was not the case (Table 4). Additionally, it could be expected that longer survival would be accompanied by larger cumulative doses of concurrent vasoconstrictor. That also could not be detected (Table 4). Thus, it appears unlikely that improved survival could have caused larger cumulative albumin doses to be administered.

The limitations of the studies assembled in this meta-analysis arguably preclude evidence-based recommendations for clinical practice. Nonetheless, this meta-analysis does furnish the best current evidence on an issue of unequivocal clinical importance: whether patient survival might be improved by albumin dose optimization. It is recognized that meta-analyses can play an important role in setting a clinical research agenda [7]. This meta-analysis suggests the need for future albumin dose-ranging studies. Furthermore, the meta-analysis provides specific guidance in the design of such studies, for instance, by identifying the range of cumulative albumin doses over which effects on survival may vary. In addition, the meta-analysis quantifies the magnitude of likely treatment effects associated with varying albumin doses and would therefore be of value in sample size computations for future studies.

The finding of a significant increase in short-term survival among type 1 HRS patients with increasing cumulative albumin dose is in accord with other studies of cirrhotic patients showing survival benefits associated with albumin administration. The first such demonstrated survival increase after albumin infusion was in patients with cirrhosis and spontaneous bacterial peritonitis (SBP) in 1999 [47]. A recent meta-analysis of randomized trials in patients with SBP confirmed that mortality was decreased by 66 % among the patients who received albumin [48]. In a meta-analysis of randomized trials among cirrhotic patients undergoing large volume paracentesis, mortality was reduced by 36 % in those who received albumin compared to those who received alternative treatments to improve circulatory function [49]. Finally, in a recent randomized trial of patients with cirrhosis and episodic hepatic encephalopathy, survival at 90 days was 69 % in those who received albumin compared with 40 % of those who received saline [50]. In summary, survival benefits of albumin have now been documented in type 1 HRS, SBP, large volume paracentesis, and hepatic encephalopathy. These results point to the versatility of albumin as first-line therapy for a range of serious complications in patients with decompensated cirrhosis.

Unlike the vasoconstrictors, adverse events attributable to albumin infusion have been reported only very infrequently in the included and other studies, and these have been related to pulmonary edema and/or circulatory overload [19, 28, 32]. Two included studies specifically reported no adverse effects related to intravenous albumin administration [22, 29]. Therefore while albumin infusion, even in high total doses, appears to be well-tolerated in type 1 HRS patients, nonetheless, monitoring of central venous pressure directly by catheterization or indirectly by vena cava ultrasound for signs of excessive cardiac preload may be a prudent precaution, especially in patients receiving higher cumulative albumin doses.

The pathophysiology of type 1 HRS is complex, but pivotal to its onset is the marked reduction of effective arterial blood volume resulting from blood volume redistribution into the splanchnic circulation and relatively insufficient cardiac output [3]. This leads to renal hypoperfusion caused by both reduced renal perfusion pressure and renal vasoconstriction resulting from increased activity of the renin-angiotensin-aldosterone system (RAAS) and the sympathetic nervous system.

The hypovolemia of type 1 HRS patients is an effective not an absolute volume deficiency. A critical amount of the circulating volume becomes pooled in the enlarged splanchnic circulation because of increased portal resistance to portal flow, excess production of a number of endogenous vasodilators, most notably nitric oxide (NO), and decreased responsiveness to endogenous vasoconstrictors in that area. Pharmacologic therapy with vasoconstrictors is thought to reverse HRS primarily by working on the splanchnic vasculature to redistribute part of the splanchnic volume back to the systemic circulation.

The infusion of hyperoncotic 20 or 25 % albumin can enhance and accelerate this redistribution by drawing fluid into the central circulatory volume because of its potent oncotic properties. Hyperoncotic albumin can expand intravascular volume from 210 to 260 % of its administered volume [51]. This small volume resuscitation is advantageous in type 1 HRS patients because they already carry a large fluid load and substantial fluid infusion could potentially lead to worsening of ascites, pleural effusion or heart failure [52, 53]. In addition, albumin administration to cirrhotic patients with ascites has been shown to result in markedly reduced plasma renin activity, aldosterone levels and muscle sympathetic nerve activity in a number of patient types with ascites [54–56].

Since the endogenous albumin of patients with decompensated cirrhosis is not only present at reduced levels, but also is functionally impaired [57], the many functions key to homeostasis that albumin performs are likely to be compromised. Among albumin’s many other properties are antioxidant, ligand-binding, immunomodulatory and detoxification functions [58]. For instance, many toxic substances present at elevated levels in decompensated liver patients such as bilirubin, endotoxin, and cytokines are bound and can be immunomodulated or detoxified by albumin. Albumin also binds to NO, which plays a crucial role in the physiopathology of HRS [59]. In a study of the effect of albumin on endotoxin removal, cytokines and NO production in patients with SBP, albumin administration significantly reduced the levels of tumor necrosis factor-a (TNF-a) and NO products in both plasma and ascitic fluid [60].

Another potential mechanism through which albumin could benefit HRS patients is amelioration of the systolic dysfunction and chronotropic incompetence that contributes to the pathogenesis of HRS [55]. Low cardiac output predicts the development of HRS [61]. Albumin infusion can increase the cardiac index in HRS patients with refractory ascites [62]. In experimental cirrhosis of rats, albumin exerted a positive cardiac inotropic effect counteracting oxidative stress- and TNF-a-induced impairment of cardiac contractility [63].

## Conclusions

Despite treatment advances with vasoconstrictor and albumin, mortality among patients with type 1 HRS remains at approximately 50 %. While clinical studies have focused on the choice and optimization of vasoconstrictor, optimization of the albumin regimen has not been pursued. This meta-analysis suggests that albumin dose optimization may improve outcome. However, further studies on the dose–response relationship between infused albumin and HRS reversal and survival in patients with type 1 HRS are both warranted and needed to fully address this question.

## Additional files

Additional file 1: **MOOSE checklist.** (PDF 72 kb) Additional file 2: **Data sets.** (PDF 164 kb)

## Abbreviations

AASLD: American Association for the Study of Liver Diseases
HRS: Hepatorenal syndrome
IAC: International Ascites Club
CI: 95 % confidence interval
NO: Nitric oxide
RAAS: Renin-angiotensin-aldosterone system
SBP: Spontaneous bacterial peritonitis
TNF-a: Tumor necrosis factor-a
