# Supplementary material for: Albumin treatment regimen for type 1 hepatorenal syndrome: a dose–response meta-analysis
Source: BMC Gastroenterol. 2015 Nov 25;15:167. doi: 10.1186/s12876-015-0389-9 (PMC4660686; doi:10.1186/s12876-015-0389-9)
Supplement: Additional file 1: — MOOSE checklist. (PDF 72 kb) [file 12876_2015_389_MOESM1_ESM.pdf]

Appendix 1: MOOSE checklist

Item	Page/Other
Reporting of background should include	
Problem definition	3
Hypothesis statement	5
Description of study outcome(s)	5
Type of exposure or intervention used	4
Type of study designs used	4
Study population	4
Reporting of search strategy should include	
Qualifications of searchers (eg, librarians and investigators)	n/a
Search strategy, including time period included in the synthesis and keywords	4
Effort to include all available studies, including contact with authors	5
Databases and registries searched	4
Search software used, name and version, including special features used (eg, explosion)	4
Use of hand searching (eg, reference lists of obtained articles)	4
List of citations located and those excluded, including justification	Fig. 1
Method of addressing articles published in languages other than English	4
Method of handling abstracts and unpublished studies	4-5
Description of any contact with authors	5
Reporting of methods should include	
Description of relevance or appropriateness of studies assembled for assessing the hypothesis to be tested	4
Rationale for the selection and coding of data (eg, sound clinical principles or convenience)	4
Documentation of how data were classified and coded (eg, multiple raters, blinding, and interrater reliability)	4
Assessment of confounding (eg, comparability of cases and controls in studies where appropriate)	4
Assessment of study quality, including blinding of quality assessors; stratification or regression on possible predictors of study results	5
Assessment of heterogeneity	5
Description of statistical methods (eg, complete description of fixed or random effects models, justification of whether the chosen models account for predictors of study results, dose-response models, or cumulative meta-analysis) in sufficient detail to be replicated	5-6
Provision of appropriate tables and graphics	27-30 and Figs. 1-4
Reporting of results should include	
Graphic summarizing individual study estimates and overall estimate	Figs. 2-3
Table giving descriptive information for each study included	28-29
Results of sensitivity testing (eg, subgroup analysis)	30
Indication of statistical uncertainty of findings	30 and Figs. 2-4
Reporting of discussion should include	
Quantitative assessment of bias (eg, publication bias)	30
Justification for exclusion (eg, exclusion of non–English-language citations)	n/a
Assessment of quality of included studies	30
Reporting of conclusions should include	
Consideration of alternative explanations for observed results	10-11
Generalization of the conclusions (ie, appropriate for the data presented and within the domain of the literature review)	10-11
Guidelines for future research	10-11
Disclosure of funding source	19

n/a, not applicable
