# Supplementary material for: Albumin treatment regimen for type 1 hepatorenal syndrome: a dose–response meta-analysis
Source: BMC Gastroenterol. 2015 Nov 25;15:167. doi: 10.1186/s12876-015-0389-9 (PMC4660686; doi:10.1186/s12876-015-0389-9)
Supplement: Additional file 2: — Data sets. (PDF 164 kb) [file 12876_2015_389_MOESM2_ESM.pdf]

Appendix 2: Data sets

```
# Study: Albumin in hepatorenal syndrome
# Date: 2015-02-17
# Analyst: Mahlon M. Wilkes, PhD
# R version: 3.0.2

# Function definitions

fC <- function(x,y=1,z="") {
  formatC(x,digits=y,format="f",big.mark=z)}

scrmol2mg <- function(x) {x/88.4}

scrmg2mol <- function(x) {x*88.4}

bilimol2mg <- function(x) {x*4/68}

pool.mean <- function(n,mean,decimal=6) {
  round(sum(n*mean)/sum(n),decimal)
}

pool.sd <- function(n,sd,decimal=6) {
  k <- length(n)
  round(sqrt(sum((n-1)*sd^2)/(sum(n)-k)),decimal)
}

z <- function(xbar,sd,n,x=1.5) {
  round(pnorm((x-xbar)/sd)*n)
}

bp2map <- function(syst,diast) {
  syst/3+diast*2/3
}

# Individual patient data of von Kalckreuth et al, 2009

baseline.7492 <- data.frame(
pt.id=as.integer(c(1,2,3,4,5,6,7,7,8,9,10,11,12,13,14,15,16,
  16,17,18,19,20,21,21,22,19,19,23,24,24,25,26,27,28,26,26,
  29,30)),
tx.n=as.integer(c(1,1,1,1,1,1,2,2,1,1,1,1,1,1,1,1,2,2,1,1,3,
  1,2,2,1,3,3,1,2,2,1,3,1,1,3,3,1,1)),
success=as.integer(c(0,0,1,1,1,0,1,1,0,1,0,1,1,1,1,0,1,1,0,
  0,3,1,0,0,1,3,3,1,2,2,1,2,1,1,2,2,1,1)),
age=as.integer(c(56,32,51,46,67,56,48,48,68,39,49,34,48,40,
  69,58,48,48,67,52,62,38,56,56,60,62,62,67,49,49,56,47,60,
  60,47,47,48,54)),
sex=c(“m”,"m","m","f","f","f","f","f","f","m","m","m","f",
  "f",“m”,"f","m","m","m","m","m","f","m","m","m","m","m",
  "f","m",“m”,"f","m","f","m","m","m","f","f"),
etiology=c(“alcoholic”,"alcoholic","alcoholic","alcoholic",
  “autoimmune”,"alcoholic","hepatitis C","hepatitis C",
  “hepatitis C”,"alcoholic","alcoholic + other","alcoholic",
  “alcoholic”,"hepatitis C","alcoholic","alcoholic",
  "alcoholic",“alcoholic”,"hepatitis B + C","alcoholic",
  "hepatitis B",“alcoholic”,"alcoholic","alcoholic",
  "hepatitis C",“hepatitis B”,"hepatitis B",
  "alcoholic + hepatitis B",“alcoholic”,"alcoholic",
  "alcoholic","alcoholic","alcoholic",“alcoholic”,
  "alcoholic","alcoholic","alcoholic","alcoholic"),
hrs.type=c(“I”,"I","I","I","I","I","II","II","II","I","I",
  "I",“I”,"I","I","I","I","I","I","I","II","I","I","I","II",
  "I",“II”,"I","I","I","I","I","I","I","I","I","I","I"),
child=as.integer(c(3,3,2,3,3,3,3,3,3,3,3,3,3,3,3,2,3,3,3,2,
  3,3,3,3,3,3,3,3,3,3,3,3,2,3,3,3,2,3)),
death=c(“y”,"n","y","y","y","y","y","y","n","y","y","y","y",
  “y”,"n","n","n","n","y","n","y","n","y","y","n","y","y",
  "n",“n”,"n","y","n","n","n","n","n","n","n"),
```

```
transplant=c(“n”,"n","n","n","n","n","y","y","y","n","n","n",
  “n”,"y","n","n","n","n","n","y","n","n","n","n","y","n",
  "n",“y”,"y","y","n","y","n","n","y","y","n","n"))

# Study level data

study <- list(

data.frame(
study.id=6602,
year=2008,
author="Sanyal et al",
design=factor(“randomized”,
  levels=c(“randomized”,"prospective","retrospective")),
hrs.def="IAC saline and/or albumin",
reversal.def="SCr<=1.5",
followup=180),

data.frame(
study.id=6601,
year=2008,
author="Martín-Llahí et al",
design="randomized",
hrs.def="IAC unspecified",
reversal.def=paste(“SCr<”,
  fC(scrmol2mg(133)),sep=""),
followup=90),

data.frame(
study.id=6739,
year=2008,
author="Neri et al",
design="randomized",
hrs.def="IAC saline",
reversal.def="SCr<1.5",
followup=180),

data.frame(
study.id=7504,
year=2002,
author="Moreau et al",
design="retrospective",
hrs.def="IAC unspecified",
reversal.def=paste(“SCr<”,
  fC(scrmol2mg(130)),sep=""),
followup=63),

data.frame(
study.id=7503,
year=2002,
author="Halimi et al",
design="retrospective",
hrs.def="IAC unspecified",
reversal.def="SCr<1.5",
followup=1080),

data.frame(
study.id=7505,
year=2001,
author="Mulkay et al",
design="prospective",
hrs.def="IAC saline+albumin",
reversal.def="SCr<1.5",
followup=95),

data.frame(
study.id=7380,
year=2009,
author="Muñoz et al",
design="prospective",
```

```
hrs.def="IAC albumin",
reversal.def="SCr<1.5",
followup=450),

data.frame(
study.id=7506,
year=2000,
author="Uriz et al",
design="prospective",
hrs.def="IAC unspecified",
reversal.def="SCr<1.5",
followup=390),

data.frame(
study.id=8255,
year=2012,
author="Narahara et al",
design="prospective",
hrs.def="IAC saline",
reversal.def="SCr<1.5",
followup=12*7),

data.frame(
study.id=7492,
year=2009,
author="von Kalckreuth et al",
design="retrospective",
hrs.def="IAC saline",
reversal.def="SCr<=1.5",
followup=NA),

data.frame(
study.id=8351,
year=2011,
author="Silawat et al",
design="randomized",
hrs.def="IAC unspecified",
reversal.def="SCr<1.5",
followup=NA),

data.frame(
study.id=7497,
year=2007,
author="Alessandria et al",
design="randomized",
hrs.def="IAC albumin",
reversal.def="SCr<1.5",
followup=180),

data.frame(
study.id=7496,
year=2008,
author="Sharma et al",
design="randomized",
hrs.def="IAC albumin",
reversal.def="SCr<=1.5",
followup=30),

data.frame(
study.id=8454,
year=2012,
author="Singh et al",
design="randomized",
hrs.def="IAC unspecified",
reversal.def="SCr<1.5",
followup=30),

data.frame(
study.id=8444,
year=2000,
```

```
author="Duhamel et al",
design="retrospective",
hrs.def="IAC unspecified",
reversal.def=paste(“SCr<”,
  fC(scrmol2mg(133)),sep=""),
followup=365),

data.frame(
study.id=7539,
year=2010,
author="Rivero et al",
design="prospective",
hrs.def="IAC unspecified",
reversal.def="SCr<1.5",
followup=90),

data.frame(
study.id=7462,
year=2009,
author="Skagen et al",
design="retrospective",
hrs.def="IAC colloid or crystalloid",
reversal.def=NA,
followup=90),

data.frame(
study.id=7542,
year=2007,
author="Esrailian et al",
design="retrospective",
hrs.def="IAC saline+albumin",
reversal.def="SCr<=1.5",
followup=360),

data.frame(
study.id=9168,
year=2012,
author="Tavakkoli et al",
design="randomized",
hrs.def="IAC albumin",
reversal.def="SCr<1.5",
followup=90),

data.frame(
study.id=7543,
year=1999,
author="Angeli et al",
design="prospective",
hrs.def="IAC saline+albumin",
reversal.def="SCr<1.5",
followup=30),

data.frame(
study.id=7518,
year=2004,
author="Wong et al",
design="prospective",
hrs.def="IAC albumin",
reversal.def=paste(“SCr<”,
  fC(scrmol2mg(135)),sep=""),
followup=90),

data.frame(
study.id=8143,
year=2011,
author="Salerno et al",
design="prospective",
hrs.def="IAC albumin",
reversal.def="SCr<1.5",
followup=90))
```

```
study <- do.call(rbind,study)

# Group level data

group <- list(

data.frame(
group.id=1,
study.id=6602,
n=56,
age=50.6,
age.sd=10.5,
scr.0=3.96,
scr.0.sd=2.19,
bilirubin=15.0,
bilirubin.sd=13.6,
salb=2.6,
salb.sd=0.84,
scr.incr.vol=NA,
ascites=54,
sbp=NA,
infection=14,
bleed.gi=2,
hcc=4,
map=75.5,
map.sd=11.4,
hr=NA,
hr.sd=NA,
hbg=NA,
hbg.sd=NA,
wbc=NA,
wbc.sd=NA,
plt=NA,
plt.sd=NA,
dose.terl=pool.mean(c(56-13,13),
  c(4,pool.mean(c(3,6.3-3),c(4,8)))),
t.terl=6.3,
dose.nadr=NA,
t.nadr=NA,
dose.mido=NA,
t.mido=NA,
dose.octreo=NA,
t.octreo=NA,
dose.alb.dx=NA,
t.alb.dx=NA,
dose.alb.tx=48.2,
t.alb.tx=6.3,
dose.alb.lvp=NA,
t.alb.lvp=NA,
dose.alb.sbp=NA,
t.alb.sbp=NA,
conc.alb=NA,
reversal=19,
t.reversal=14,
survival=24,
t.survival=180),

data.frame(
group.id=1,
study.id=6601,
n=17,
age=59,
age.sd=10,
scr.0=scrmol2mg(318.2),
scr.0.sd=scrmol2mg(128.8),
bilirubin=bilimol2mg(307.8),
bilirubin.sd=bilimol2mg(324.9),
salb=3.0,
salb.sd=0.7,
scr.incr.vol=NA,
```

```
ascites=NA,
sbp=NA,
infection=12,
bleed.gi=NA,
hcc=NA,
map=73,
map.sd=10,
hr=NA,
hr.sd=NA,
hbg=NA,
hbg.sd=NA,
wbc=NA,
wbc.sd=NA,
plt=NA,
plt.sd=NA,
dose.terl=pool.mean(c(3,4),c(6,12)),
t.terl=7,
dose.nadr=NA,
t.nadr=NA,
dose.mido=NA,
t.mido=NA,
dose.octreo=NA,
t.octreo=NA,
dose.alb.dx=NA,
t.alb.dx=NA,
dose.alb.tx=190/7,
t.alb.tx=7,
dose.alb.lvp=NA,
t.alb.lvp=NA,
dose.alb.sbp=NA,
t.alb.sbp=NA,
conc.alb=20,
reversal=z(115,18,6,scrmg2mol(1.5)),
t.reversal=15,
survival=NA,
t.survival=90),

data.frame(
group.id=1,
study.id=6739,
n=26,
age=59,
age.sd=4,
scr.0=scrmol2mg(248),
scr.0.sd=scrmol2mg(96),
bilirubin=NA,
bilirubin.sd=NA,
salb=2.71,
salb.sd=0.32,
scr.incr.vol=NA,
ascites=26,
sbp=NA,
infection=5,
bleed.gi=0,
hcc=0,
map=82,
map.sd=2,
hr=85.35,
hr.sd=9.4,
hbg=NA,
hbg.sd=NA,
wbc=NA,
wbc.sd=NA,
plt=NA,
plt.sd=NA,
dose.terl=pool.mean(c(5,14),c(3,1.5)),
t.terl=5+14,
dose.nadr=NA,
t.nadr=NA,
dose.mido=NA,
t.mido=NA,
dose.octreo=NA,
t.octreo=NA,
dose.alb.dx=NA,
t.alb.dx=NA,
dose.alb.tx=44,
t.alb.tx=5+14,
dose.alb.lvp=NA,
t.alb.lvp=NA,
dose.alb.sbp=NA,
t.alb.sbp=NA,
conc.alb=20,
reversal=21,
t.reversal=14,
survival=26-10,
t.survival=180),

data.frame(
group.id=1,
study.id=7504,
n=99,
age=56,
age.sd=10,
scr.0=scrmol2mg(254),
scr.0.sd=scrmol2mg(99),
bilirubin=bilimol2mg(200),
bilirubin.sd=bilimol2mg(220),
salb=2.75,
salb.sd=0.73,
scr.incr.vol=NA,
ascites=99,
sbp=NA,
infection=36,
bleed.gi=22,
hcc=10,
map=78,
map.sd=20,
hr=80,
hr.sd=18,
hbg=NA,
hbg.sd=NA,
wbc=NA,
wbc.sd=NA,
plt=NA,
plt.sd=NA,
dose.terl=3.2,
t.terl=11.4,
dose.nadr=NA,
t.nadr=NA,
dose.mido=NA,
t.mido=NA,
dose.octreo=NA,
t.octreo=NA,
dose.alb.dx=NA,
t.alb.dx=NA,
dose.alb.tx=38,
t.alb.tx=11.4,
dose.alb.lvp=NA,
t.alb.lvp=NA,
dose.alb.sbp=NA,
t.alb.sbp=NA,
conc.alb=NA,
reversal=z(138,59,58,scrmg2mol(1.5)),
t.reversal=11,
survival=99-75,
t.survival=360),

data.frame(
group.id=1,
study.id=7503,
```

```
n=16,
age=60.4,
age.sd=9.8,
scr.0=scrmol2mg(286),
scr.0.sd=scrmol2mg(112),
bilirubin=bilimol2mg(196),
bilirubin.sd=bilimol2mg(186),
salb=2.6,
salb.sd=0.7,
scr.incr.vol=NA,
ascites=NA,
sbp=NA,
infection=NA,
bleed.gi=NA,
hcc=0,
map=bp2map(123,62),
map.sd=bp2map(15,14),
hr=NA,
hr.sd=NA,
hbg=NA,
hbg.sd=NA,
wbc=NA,
wbc.sd=NA,
plt=NA,
plt.sd=NA,
dose.terl=4,
t.terl=7,
dose.nadr=NA,
t.nadr=NA,
dose.mido=NA,
t.mido=NA,
dose.octreo=NA,
t.octreo=NA,
dose.alb.dx=NA,
t.alb.dx=NA,
dose.alb.tx=0,
t.alb.tx=0,
dose.alb.lvp=NA,
t.alb.lvp=NA,
dose.alb.sbp=NA,
t.alb.sbp=NA,
conc.alb=NA,
reversal=6,
t.reversal=5,
survival=1,
t.survival=1080),

data.frame(
group.id=1,
study.id=7505,
n=12,
age=53.5,
age.sd=(60.2-44.8)/3,
scr.0=3.4,
scr.0.sd=(4.0-2.5)/3,
bilirubin=6.2,
bilirubin.sd=(20.5-1.7)/3,
salb=2.9,
salb.sd=(3.4-2.4)/3,
scr.incr.vol=NA,
ascites=12,
sbp=NA,
infection=NA,
bleed.gi=NA,
hcc=3,
map=76,
map.sd=(83-68)/3,
hr=91,
hr.sd=(110-74)/3,
hbg=NA,
hbg.sd=NA,
wbc=NA,
wbc.sd=NA,
plt=NA,
plt.sd=NA,
dose.terl=pool.mean(c(10,16),c(4,2)),
t.terl=26,
dose.nadr=NA,
t.nadr=NA,
dose.mido=NA,
t.mido=NA,
dose.octreo=NA,
t.octreo=NA,
dose.alb.dx=60,
t.alb.dx=2,
dose.alb.tx=pool.mean(c(4,2),c(40,20)),
t.alb.tx=6,
dose.alb.lvp=NA,
t.alb.lvp=NA,
dose.alb.sbp=NA,
t.alb.sbp=NA,
conc.alb=NA,
reversal=5,
t.reversal=26,
survival=12-9,
t.survival=95),

data.frame(
group.id=1,
study.id=7380,
n=13,
age=pool.mean(c(8,5),c(51.5,58.6)),
age.sd=pool.sd(c(8.5),c(5.3*sqrt(8),6.9*sqrt(5))),
scr.0=pool.mean(c(8,5),c(3.0,3.9)),
scr.0.sd=pool.sd(c(8.5),c(1.7*sqrt(8),1.5*sqrt(5))),
bilirubin=NA,
bilirubin.sd=NA,
salb=NA,
salb.sd=NA,
scr.incr.vol=NA,
ascites=NA,
sbp=NA,
infection=1,
bleed.gi=2,
hcc=NA,
map=pool.mean(c(8,5),c(70.1,68.8)),
map.sd=pool.sd(c(8.5),c(9.1*sqrt(8),6.5*sqrt(5))),
hr=NA,
hr.sd=NA,
hbg=NA,
hbg.sd=NA,
wbc=NA,
wbc.sd=NA,
plt=NA,
plt.sd=NA,
dose.terl=pool.mean(c(8,5),c(55.6/12.5,20.2/5)),
t.terl=pool.mean(c(8,5),c(12.5,5)),
dose.nadr=NA,
t.nadr=NA,
dose.mido=NA,
t.mido=NA,
dose.octreo=NA,
t.octreo=NA,
dose.alb.dx=mean(c(30,80)),
t.alb.dx=1.5,
dose.alb.tx=pool.mean(c(8,5),c(57.5,68)),
t.alb.tx=pool.mean(c(8,5),c(12.5,5)),
dose.alb.lvp=NA,
t.alb.lvp=NA,
dose.alb.sbp=NA,
```

```
t.alb.sbp=NA,
conc.alb=NA,
reversal=z(1.7,0.8*sqrt(8),8),
t.reversal=9,
survival=3,
t.survival=450),

data.frame(
group.id=1,
study.id=7506,
n=6,
age=54,
age.sd=(75-42)/3,
scr.0=3.9,
scr.0.sd=0.7*sqrt(9),
bilirubin=14,
bilirubin.sd=6*sqrt(9),
salb=3.2,
salb.sd=0.2*sqrt(9),
scr.incr.vol=NA,
ascites=6,
sbp=NA,
infection=NA,
bleed.gi=NA,
hcc=NA,
map=68,
map.sd=2*sqrt(9),
hr=81,
hr.sd=4*sqrt(9),
hbg=NA,
hbg.sd=NA,
wbc=NA,
wbc.sd=NA,
plt=NA,
plt.sd=NA,
dose.terl=0.75*6,
t.terl=10.6,
dose.nadr=NA,
t.nadr=NA,
dose.mido=NA,
t.mido=NA,
dose.octreo=NA,
t.octreo=NA,
dose.alb.dx=NA,
t.alb.dx=NA,
dose.alb.tx=pool.mean(c(1,10.6-1),c(70,30)),
t.alb.tx=10.6,
dose.alb.lvp=NA,
t.alb.lvp=NA,
dose.alb.sbp=NA,
t.alb.sbp=NA,
conc.alb=20,
reversal=5,
t.reversal=15,
survival=NA,
t.survival=NA),
#For reversal Fig. 1, SCr>2.4 (see text, p. 45)

data.frame(
group.id=1,
study.id=8255,
n=8,
age=59.1,
age.sd=11.8,
scr.0=3.01,
scr.0.sd=0.76,
bilirubin=9.4,
bilirubin.sd=7.6,
salb=2.5,
salb.sd=0.4,
scr.incr.vol=NA,
ascites=8,
sbp=NA,
infection=4,
bleed.gi=1,
hcc=0,
map=74,
map.sd=14,
hr=94,
hr.sd=8,
hbg=NA,
hbg.sd=NA,
wbc=NA,
wbc.sd=NA,
plt=NA,
plt.sd=NA,
dose.terl=2.8,
t.terl=6.3,
dose.nadr=NA,
t.nadr=NA,
dose.mido=NA,
t.mido=NA,
dose.octreo=NA,
t.octreo=NA,
dose.alb.dx=NA,
t.alb.dx=NA,
dose.alb.tx=25.7,
t.alb.tx=6.3,
dose.alb.lvp=NA,
t.alb.lvp=NA,
dose.alb.sbp=NA,
t.alb.sbp=NA,
conc.alb=NA,
reversal=6,
t.reversal=15,
survival=1,
t.survival=84),

data.frame(
group.id=1,
study.id=7492,
n=30-6,
age=mean(baseline.7492[baseline.7492$hrs.typ=="I","age"]),
age.sd=sd(baseline.7492[baseline.7492$hrs.typ=="I","age"]),
scr.0=NA,
scr.0.sd=NA,
bilirubin=NA,
bilirubin.sd=NA,
salb=NA,
salb.sd=NA,
scr.incr.vol=NA,
ascites=NA,
sbp=NA,
infection=NA,
bleed.gi=NA,
hcc=NA,
map=NA,
map.sd=NA,
hr=NA,
hr.sd=NA,
hbg=NA,
hbg.sd=NA,
wbc=NA,
wbc.sd=NA,
plt=NA,
plt.sd=NA,
dose.terl=pool.mean(c(25,13),c(3.9,3.4)),
t.terl=pool.mean(c(25,13),c(6.5,8.3)),
dose.nadr=NA,
t.nadr=NA,
```

```
dose.mido=NA,
t.mido=NA,
dose.octreo=NA,
t.octreo=NA,
dose.alb.dx=NA,
t.alb.dx=NA,
dose.alb.tx=pool.mean(c(25,13),c(28.5,24.6)),
t.alb.tx=pool.mean(c(25,13),c(6.5,8.3)),
dose.alb.lvp=NA,
t.alb.lvp=NA,
dose.alb.sbp=NA,
t.alb.sbp=NA,
conc.alb=NA,
reversal=30-6-9,
t.reversal=20,
survival=17,
t.survival=NA),

data.frame(
group.id=1,
study.id=8351,
n=30,
age=NA,
age.sd=NA,
scr.0=3.01,
scr.0.sd=1.255,
bilirubin=3.40,
bilirubin.sd=2.092,
salb=2.45,
salb.sd=0.673,
scr.incr.vol=NA,
ascites=NA,
sbp=NA,
infection=NA,
bleed.gi=NA,
hcc=NA,
map=bp2map(92.3,55.3),
map.sd=bp2map(19.77,14.79),
hr=NA,
hr.sd=NA,
hbg=NA,
hbg.sd=NA,
wbc=NA,
wbc.sd=NA,
plt=NA,
plt.sd=NA,
dose.terl=1.5,
t.terl=7,
dose.nadr=NA,
t.nadr=NA,
dose.mido=NA,
t.mido=NA,
dose.octreo=NA,
t.octreo=NA,
dose.alb.dx=NA,
t.alb.dx=NA,
dose.alb.tx=12.5,
t.alb.tx=7,
dose.alb.lvp=NA,
t.alb.lvp=NA,
dose.alb.sbp=NA,
t.alb.sbp=NA,
conc.alb=NA,
reversal=round(30*pnorm((1.5-1.34)/0.554)),
t.reversal=7,
survival=30-6,
t.survival=NA),

data.frame(
group.id=1,
study.id=7497,
n=5,
age=55,
age.sd=2*sqrt(12),
scr.0=2.5,
scr.0.sd=0.3*sqrt(12),
bilirubin=5.1,
bilirubin.sd=1*sqrt(12),
salb=3,
salb.sd=0.1*sqrt(12),
scr.incr.vol=NA,
ascites=NA,
sbp=NA,
infection=NA,
bleed.gi=NA,
hcc=NA,
map=74,
map.sd=3*sqrt(12),
hr=NA,
hr.sd=NA,
hbg=NA,
hbg.sd=NA,
wbc=NA,
wbc.sd=NA,
plt=NA,
plt.sd=NA,
dose.terl=pool.mean(c(8,2),c(6,12)),
t.terl=pool.mean(c(4,1),c(6,14)),
dose.nadr=NA,
t.nadr=NA,
dose.mido=NA,
t.mido=NA,
dose.octreo=NA,
t.octreo=NA,
dose.alb.dx=NA,
t.alb.dx=NA,
dose.alb.tx=46,
t.alb.tx=pool.mean(c(4,1),c(6,14)),
dose.alb.lvp=NA,
t.alb.lvp=NA,
dose.alb.sbp=NA,
t.alb.sbp=NA,
conc.alb=20,
reversal=4,
t.reversal=pool.mean(c(4,1),c(6,14)),
survival=5-1,
t.survival=180),

data.frame(
group.id=2,
study.id=7497,
n=4,
age=56,
age.sd=3*sqrt(10),
scr.0=2.3,
scr.0.sd=0.2*sqrt(10),
bilirubin=4.1,
bilirubin.sd=1*sqrt(10),
salb=3,
salb.sd=0.2*sqrt(10),
scr.incr.vol=NA,
ascites=NA,
sbp=NA,
infection=NA,
bleed.gi=NA,
hcc=NA,
map=71,
map.sd=2*sqrt(10),
hr=NA,
hr.sd=NA,
```

```
hbg=NA,
hbg.sd=NA,
wbc=NA,
wbc.sd=NA,
plt=NA,
plt.sd=NA,
dose.terl=NA,
t.terl=NA,
dose.nadr=0.3*60*60*24/1000,
t.nadr=pool.mean(c(3,1),c(5,14)),
dose.mido=NA,
t.mido=NA,
dose.octreo=NA,
t.octreo=NA,
dose.alb.dx=NA,
t.alb.dx=NA,
dose.alb.tx=56,
t.alb.tx=pool.mean(c(3,1),c(5,14)),
dose.alb.lvp=NA,
t.alb.lvp=NA,
dose.alb.sbp=NA,
t.alb.sbp=NA,
conc.alb=20,
reversal=3,
t.reversal=pool.mean(c(3,1),c(5,14)),
survival=4-2,
t.survival=180),

data.frame(
group.id=1,
study.id=7496,
n=20,
age=47.8,
age.sd=9.8,
scr.0=3.0,
scr.0.sd=0.5,
bilirubin=7.6,
bilirubin.sd=(40.0-0.7)/4,
salb=2.6,
salb.sd=0.6,
scr.incr.vol=NA,
ascites=NA,
sbp=NA,
infection=NA,
bleed.gi=NA,
hcc=NA,
map=81.4,
map.sd=11.4,
hr=NA,
hr.sd=NA,
hbg=NA,
hbg.sd=NA,
wbc=NA,
wbc.sd=NA,
plt=NA,
plt.sd=NA,
dose.terl=5.8,
t.terl=8.1,
dose.nadr=NA,
t.nadr=NA,
dose.mido=NA,
t.mido=NA,
dose.octreo=NA,
t.octreo=NA,
dose.alb.dx=60,
t.alb.dx=2,
dose.alb.tx=30,
t.alb.tx=8.1,
dose.alb.lvp=NA,
t.alb.lvp=NA,
dose.alb.sbp=NA,
t.alb.sbp=NA,
conc.alb=20,
reversal=8,
t.reversal=15,
survival=11,
t.survival=30),

data.frame(
group.id=2,
study.id=7496,
n=20,
age=48.2,
age.sd=13.4,
scr.0=3.3,
scr.0.sd=1.3,
bilirubin=5.2,
bilirubin.sd=(28.0-1.0)/4,
salb=2.4,
salb.sd=0.4,
scr.incr.vol=NA,
ascites=NA,
sbp=NA,
infection=NA,
bleed.gi=NA,
hcc=NA,
map=78.2,
map.sd=5.3,
hr=NA,
hr.sd=NA,
hbg=NA,
hbg.sd=NA,
wbc=NA,
wbc.sd=NA,
plt=NA,
plt.sd=NA,
dose.terl=NA,
t.terl=NA,
dose.nadr=1*24,
t.nadr=7.8,
dose.mido=NA,
t.mido=NA,
dose.octreo=NA,
t.octreo=NA,
dose.alb.dx=60,
t.alb.dx=2,
dose.alb.tx=30,
t.alb.tx=7.8,
dose.alb.lvp=NA,
t.alb.lvp=NA,
dose.alb.sbp=NA,
t.alb.sbp=NA,
conc.alb=20,
reversal=10,
t.reversal=15,
survival=11,
t.survival=30),

data.frame(
group.id=1,
study.id=8454,
n=23,
age=51.4,
age.sd=11.6,
scr.0=3.27,
scr.0.sd=0.71,
bilirubin=3.99,
bilirubin.sd=2.58,
salb=2.78,
salb.sd=0.40,
```

```
scr.incr.vol=NA,
ascites=23,
sbp=NA,
infection=7,
bleed.gi=NA,
hcc=NA,
map=64.7,
map.sd=11.9,
hr=NA,
hr.sd=NA,
hbg=NA,
hbg.sd=NA,
wbc=NA,
wbc.sd=NA,
plt=NA,
plt.sd=NA,
dose.terl=3.13,
t.terl=7.82,
dose.nadr=NA,
t.nadr=NA,
dose.mido=NA,
t.mido=NA,
dose.octreo=NA,
t.octreo=NA,
dose.alb.dx=NA,
t.alb.dx=NA,
dose.alb.tx=20,
t.alb.tx=7.82,
dose.alb.lvp=NA,
t.alb.lvp=NA,
dose.alb.sbp=NA,
t.alb.sbp=NA,
conc.alb=NA,
reversal=9,
t.reversal=15,
survival=7,
t.survival=30),

data.frame(
group.id=2,
study.id=8454,
n=23,
age=48.3,
age.sd=11.6,
scr.0=3.10,
scr.0.sd=0.66,
bilirubin=4.66,
bilirubin.sd=5.72,
salb=2.78,
salb.sd=0.20,
scr.incr.vol=NA,
ascites=23,
sbp=NA,
infection=6,
bleed.gi=NA,
hcc=NA,
map=65.2,
map.sd=10.2,
hr=NA,
hr.sd=NA,
hbg=NA,
hbg.sd=NA,
wbc=NA,
wbc.sd=NA,
plt=NA,
plt.sd=NA,
dose.terl=NA,
t.terl=NA,
dose.nadr=0.59*24,
t.nadr=9.3,
dose.mido=NA,
t.mido=NA,
dose.octreo=NA,
t.octreo=NA,
dose.alb.dx=NA,
t.alb.dx=NA,
dose.alb.tx=20,
t.alb.tx=9.3,
dose.alb.lvp=NA,
t.alb.lvp=NA,
dose.alb.sbp=NA,
t.alb.sbp=NA,
conc.alb=NA,
reversal=10,
t.reversal=15,
survival=8,
t.survival=30),

data.frame(
group.id=1,
study.id=8444,
n=12,
age=54.5,
age.sd=(75-40)/3,
scr.0=scrmol2mg(mean(c(130.9,176.4,196.0,199.6,204.2,
  221.4,246.9,265.5,274.2,278.3,338.8,381.9))),
scr.0.sd=scrmol2mg(sd(c(130.9,176.4,196.0,199.6,204.2,
  221.4,246.9,265.5,274.2,278.3,338.8,381.9))),
bilirubin=NA,
bilirubin.sd=NA,
salb=NA,
salb.sd=NA,
scr.incr.vol=NA,
ascites=12,
sbp=NA,
infection=4,
bleed.gi=NA,
hcc=NA,
map=NA,
map.sd=NA,
hr=NA,
hr.sd=NA,
hbg=NA,
hbg.sd=NA,
wbc=NA,
wbc.sd=NA,
plt=NA,
plt.sd=NA,
dose.terl=mean(c(2,6)),
t.terl=mean(c(2,4,4,8,9,10)),
dose.nadr=NA,
t.nadr=NA,
dose.mido=NA,
t.mido=NA,
dose.octreo=NA,
t.octreo=NA,
dose.alb.dx=NA,
t.alb.dx=NA,
dose.alb.tx=0,
t.alb.tx=0,
dose.alb.lvp=NA,
t.alb.lvp=NA,
dose.alb.sbp=NA,
t.alb.sbp=NA,
conc.alb=NA,
reversal=6,
t.reversal=10,
survival=12-8,
t.survival=NA),
```

```
data.frame(
group.id=1,
study.id=7539,
n=41,
age=NA,
age.sd=NA,
scr.0=NA,
scr.0.sd=NA,
bilirubin=NA,
bilirubin.sd=NA,
salb=NA,
salb.sd=NA,
scr.incr.vol=NA,
ascites=NA,
sbp=NA,
infection=20,
bleed.gi=NA,
hcc=NA,
map=NA,
map.sd=NA,
hr=NA,
hr.sd=NA,
hbg=NA,
hbg.sd=NA,
wbc=NA,
wbc.sd=NA,
plt=NA,
plt.sd=NA,
dose.terl=1.5*6,
t.terl=7,
dose.nadr=NA,
t.nadr=NA,
dose.mido=NA,
t.mido=NA,
dose.octreo=NA,
t.octreo=NA,
dose.alb.dx=NA,
t.alb.dx=NA,
dose.alb.tx=mean(c(70,rep(30,6))),
t.alb.tx=7,
dose.alb.lvp=NA,
t.alb.lvp=NA,
dose.alb.sbp=NA,
t.alb.sbp=NA,
conc.alb=NA,
reversal=22,
t.reversal=NA,
survival=round(0.55*20)+round(0.24*21),
t.survival=90),

data.frame(
group.id=1,
study.id=7462,
n=49,
age=52.73,
age.sd=10.56,
scr.0=2.50,
scr.0.sd=1.26,
bilirubin=NA,
bilirubin.sd=NA,
salb=NA,
salb.sd=NA,
scr.incr.vol=NA,
ascites=round(49*97/102),
sbp=NA,
infection=round(49*27/102),
bleed.gi=NA,
hcc=NA,
map=NA,
map.sd=NA,
hr=NA,
hr.sd=NA,
hbg=NA,
hbg.sd=NA,
wbc=NA,
wbc.sd=NA,
plt=NA,
plt.sd=NA,
dose.terl=NA,
t.terl=NA,
dose.nadr=NA,
t.nadr=NA,
dose.mido=8.8*3,
t.mido=8.4,
dose.octreo=0.142*3,
t.octreo=8.4,
dose.alb.dx=NA,
t.alb.dx=NA,
dose.alb.tx=43.8,
t.alb.tx=8.4,
dose.alb.lvp=NA,
t.alb.lvp=NA,
dose.alb.sbp=NA,
t.alb.sbp=NA,
conc.alb=NA,
reversal=NA,
t.reversal=30,
survival=round(0.44*49),
t.survival=90),

data.frame(
group.id=1,
study.id=7542,
n=60,
age=46.6,
age.sd=NA,
scr.0=2.1,
scr.0.sd=NA,
bilirubin=15.2,
bilirubin.sd=NA,
salb=1.9,
salb.sd=NA,
scr.incr.vol=NA,
ascites=round(0.95*60),
sbp=NA,
infection=round(0.22*60),
bleed.gi=round(0.48*60),
hcc=NA,
map=NA,
map.sd=NA,
hr=NA,
hr.sd=NA,
hbg=NA,
hbg.sd=NA,
wbc=NA,
wbc.sd=NA,
plt=123,
plt.sd=NA,
dose.terl=NA,
t.terl=NA,
dose.nadr=NA,
t.nadr=NA,
dose.mido=3*pool.mean(c(3,28,9,11,8),seq(5,15,by=2.5)),
t.mido=16.8,
dose.octreo=3*pool.mean(c(23,35),c(100,200)),
t.octreo=16.8,
dose.alb.dx=60,
t.alb.dx=2,
dose.alb.tx=0,
t.alb.tx=0,
```

```
dose.alb.lvp=NA,
t.alb.lvp=NA,
dose.alb.sbp=NA,
t.alb.sbp=NA,
conc.alb=NA,
reversal=24,
t.reversal=30,
survival=60-26,
t.survival=30),

data.frame(
group.id=1,
study.id=9168,
n=6,
age=52,
age.sd=12.95,
scr.0=2.64,
scr.0.sd=0.71,
bilirubin=7.95,
bilirubin.sd=7.76,
salb=2.67,
salb.sd=0.20,
scr.incr.vol=NA,
ascites=NA,
sbp=NA,
infection=0,
bleed.gi=NA,
hcc=0,
map=73.36,
map.sd=6.68,
hr=89.09,
hr.sd=13.04,
hbg=NA,
hbg.sd=NA,
wbc=NA,
wbc.sd=NA,
plt=NA,
plt.sd=NA,
dose.terl=NA,
t.terl=NA,
dose.nadr=0.1*56.18*60*24/1000,
t.nadr=18,
dose.mido=NA,
t.mido=NA,
dose.octreo=NA,
t.octreo=NA,
dose.alb.dx=NA,
t.alb.dx=NA,
dose.alb.tx=mean(c(20,60)),
t.alb.tx=18,
dose.alb.lvp=NA,
t.alb.lvp=NA,
dose.alb.sbp=NA,
t.alb.sbp=NA,
conc.alb=20,
reversal=3,
t.reversal=NA,
survival=2,
t.survival=90),

data.frame(
group.id=2,
study.id=9168,
n=9,
age=52.9,
age.sd=12.61,
scr.0=2.58,
scr.0.sd=0.83,
bilirubin=11.61,
bilirubin.sd=12.21,
salb=2.62,
salb.sd=0.32,
scr.incr.vol=NA,
ascites=NA,
sbp=NA,
infection=0,
bleed.gi=NA,
hcc=0,
map=69.75,
map.sd=7.08,
hr=84.25,
hr.sd=9.7,
hbg=NA,
hbg.sd=NA,
wbc=NA,
wbc.sd=NA,
plt=NA,
plt.sd=NA,
dose.terl=NA,
t.terl=NA,
dose.nadr=NA,
t.nadr=NA,
dose.mido=5*3,
t.mido=18,
dose.octreo=0.1*3,
t.octreo=18,
dose.alb.dx=NA,
t.alb.dx=NA,
dose.alb.tx=mean(c(20,60)),
t.alb.tx=18,
dose.alb.lvp=NA,
t.alb.lvp=NA,
dose.alb.sbp=NA,
t.alb.sbp=NA,
conc.alb=20,
reversal=7,
t.reversal=NA,
survival=5,
t.survival=90),

data.frame(
group.id=1,
study.id=7543,
n=5,
age=62,
age.sd=round(3*sqrt(5),1),
scr.0=5.0,
scr.0.sd=round(0.9*sqrt(5),1),
bilirubin=4.3,
bilirubin.sd=round(1.3*sqrt(5),1),
salb=3.0,
salb.sd=round(0.1*sqrt(5),1),
scr.incr.vol=5,
ascites=5,
sbp=0,
infection=0,
bleed.gi=0,
hcc=0,
map=75.9,
map.sd=round(3.0*sqrt(5),1),
hr=84,
hr.sd=round(6*sqrt(5)),
hbg=NA,
hbg.sd=NA,
wbc=NA,
wbc.sd=NA,
plt=NA,
plt.sd=NA,
dose.terl=NA,
t.terl=NA,
```

```
dose.nadr=NA,
t.nadr=NA,
dose.mido=pool.mean(c(2,3),3*c(7.5,12.5)),
t.mido=20,
dose.octreo=pool.mean(c(2,3),3*c(0.1,0.2)),
t.octreo=20,
dose.alb.dx=40,
t.alb.dx=4,
dose.alb.tx=mean(c(10,20)),
t.alb.tx=20,
dose.alb.lvp=2*3*8/5,
t.alb.lvp=1,
dose.alb.sbp=NA,
t.alb.sbp=NA,
conc.alb=20,
reversal=0,
t.reversal=20,
survival=4,
t.survival=30),

data.frame(
group.id=1,
study.id=7518,
n=14,
age=pool.mean(c(14,4),c(55.9,52.7)),
age.sd=pool.sd(c(14,4),c(2.3,5.0)*sqrt(c(10,4))),
scr.0=pool.mean(c(14,4),scrmol2mg(c(233,345))),
scr.0.sd=pool.sd(c(14,4),scrmol2mg(c(29,83)*sqrt(c(10,4)))),
bilirubin=pool.mean(c(14,4),bilimol2mg(c(45,71))),
bilirubin.sd=
  pool.sd(c(14,4),bilimol2mg(c(8,16)*sqrt(c(10,4)))),
salb=pool.mean(c(14,4),c(3.2,3.3)),
salb.sd=pool.sd(c(14,4),c(0.3,0.5)*sqrt(c(10,4))),
scr.incr.vol=NA,
ascites=14,
sbp=3,
infection=2,
bleed.gi=2,
hcc=NA,
map=pool.mean(c(14,4),c(81,79)),
map.sd=pool.sd(c(14,4),c(5,4)*sqrt(c(10,4))),
hr=pool.mean(c(14,4),c(75,91)),
hr.sd=pool.sd(c(14,4),c(5,3)*sqrt(c(10,4))),
hbg=pool.mean(c(14,4),c(9.4,9.7)),
hbg.sd=pool.sd(c(14,4),c(0.4,0.4)*sqrt(c(10,4))),
wbc=NA,
wbc.sd=NA,
plt=pool.mean(c(14,4),c(102,78)),
plt.sd=pool.sd(c(14,4),c(19,22)*sqrt(c(10,4))),
dose.terl=NA,
t.terl=NA,
dose.nadr=NA,
t.nadr=NA,
dose.mido=2.5,
t.mido=14,
dose.octreo=0.025*24,
t.octreo=14,
dose.alb.dx=50,
t.alb.dx=5,
dose.alb.tx=50,
t.alb.tx=14,
dose.alb.lvp=NA,
t.alb.lvp=NA,
dose.alb.sbp=NA,
t.alb.sbp=NA,
conc.alb=NA,
reversal=10,
t.reversal=14,
survival=14-7,
t.survival=90),
```

```
data.frame(
group.id=1,
study.id=8143,
n=40,
age=62,
age.sd=1.2*sqrt(40),
scr.0=3.17,
scr.0.sd=0.19*sqrt(40),
bilirubin=15.1,
bilirubin.sd=1.6*sqrt(40),
salb=2.8,
salb.sd=0.06*sqrt(40),
scr.incr.vol=NA,
ascites=round(40*0.96),
sbp=NA,
infection=round(40*(0.15+0.237)),
bleed.gi=round(40*0.132),
hcc=NA,
map=80.4,
map.sd=1.3*sqrt(40),
hr=76,
hr.sd=1.3*sqrt(40),
hbg=10.5,
hbg.sd=0.2*sqrt(40),
wbc=11394,
wbc.sd=848*sqrt(40),
plt=120,
plt.sd=7.4*sqrt(40),
dose.terl=pool.mean(c(20,20),c(0.5*5,12)),
t.terl=8.7,
dose.nadr=NA,
t.nadr=NA,
dose.mido=NA,
t.mido=NA,
dose.octreo=NA,
t.octreo=NA,
dose.alb.dx=mean(c(70,100)),
t.alb.dx=2,
dose.alb.tx=27,
t.alb.tx=8.7,
dose.alb.lvp=NA,
t.alb.lvp=NA,
dose.alb.sbp=NA,
t.alb.sbp=NA,
conc.alb=NA,
reversal=round(40*0.3),
t.reversal=14,
survival=NA,
t.survival=NA),

data.frame(
group.id=2,
study.id=8143,
n=24,
age=62,
age.sd=1.2*sqrt(24),
scr.0=3.17,
scr.0.sd=0.19*sqrt(24),
bilirubin=15.1,
bilirubin.sd=1.6*sqrt(24),
salb=2.8,
salb.sd=0.06*sqrt(24),
scr.incr.vol=NA,
ascites=round(24*0.96),
sbp=NA,
infection=round(24*(0.15+0.237)),
bleed.gi=round(24*0.132),
hcc=NA,
map=80.4,
map.sd=1.3*sqrt(24),
```

```
hr=76,
hr.sd=1.3*sqrt(24),
hbg=10.5,
hbg.sd=0.2*sqrt(24),
wbc=11394,
wbc.sd=848*sqrt(24),
plt=120,
plt.sd=7.4*sqrt(24),
dose.terl=NA,
t.terl=NA,
dose.nadr=NA,
t.nadr=NA,
dose.mido=pool.mean(c(22,2),c(22.5,37.5)),
t.mido=8.7,
dose.octreo=pool.mean(c(22,2),c(0.3,0.6)),
t.octreo=8.7,
dose.alb.dx=mean(c(70,100)),
t.alb.dx=2,
dose.alb.tx=27,
t.alb.tx=8.7,
dose.alb.lvp=NA,
t.alb.lvp=NA,
dose.alb.sbp=NA,
t.alb.sbp=NA,
conc.alb=NA,
reversal=round(24*0.3),
t.reversal=NA,
survival=NA,
t.survival=NA))

group <- do.call(rbind,group)

# Intermediate computations

group$dose.alb.tot <-
  ifelse(is.na(group$dose.alb.dx),
    group$dose.alb.tx,
    (group$t.alb.dx*group$dose.alb.dx+
    group$t.alb.tx*group$dose.alb.tx)/
    (group$t.alb.dx+group$t.alb.tx))

group$t.alb.tot <-
  ifelse(is.na(group$t.alb.dx),
    group$t.alb.tx,
    group$t.alb.dx+group$t.alb.tx)

group$dose.alb.cum <-
  group$t.alb.tx*group$dose.alb.tx

study <-
  study[!study$study.id%in%group[group$dose.alb.cum==0,"study
  .id"],]

row.names(study) <- NULL

group <- group[group$dose.alb.cum>0,]

study <- merge(study,
  data.frame(
    study.id=as.numeric(unlist(dimnames(
    tapply(group$n,group$study.id,sum)))),
    N=tapply(group$n,group$study.id,sum)))

study <- study[order(study$year,study$author),]

row.names(study) <- NULL

group$terl.z <-
  (group$dose.terl*group$t.terl-
  mean(group$dose.terl*group$t.terl,na.rm=TRUE))/
  sd(group$dose.terl*group$t.terl,na.rm=TRUE)

group$mido.z <-
  (group$dose.mido*group$t.mido-
  mean(group$dose.mido*group$t.mido,na.rm=TRUE))/
  sd(group$dose.mido*group$t.mido,na.rm=TRUE)

group$octreo.z <-
  (group$dose.octreo*group$t.octreo-
  mean(group$dose.octreo*group$t.octreo,na.rm=TRUE))/
  sd(group$dose.octreo*group$t.octreo,na.rm=TRUE)

group$nadr.z <-
  (group$dose.nadr*group$t.nadr-
  mean(group$dose.nadr*group$t.nadr,na.rm=TRUE))/
  sd(group$dose.nadr*group$t.nadr,na.rm=TRUE)

group$vaso.z <- (group$mido.z+group$octreo.z)/2

group[!is.na(group$dose.terl),"vaso.z"] <-
  group[!is.na(group$dose.terl),"terl.z"]

group[!is.na(group$dose.nadr),"vaso.z"] <-
  group[!is.na(group$dose.nadr),"nadr.z"]

group$drug <-
  factor(ifelse(!is.na(group$dose.terl),
    “terlipressin”,
    ifelse(!is.na(group$dose.nadr),
      “noradrenaline”,"midodrine/octreotide")),
      levels=c(“terlipressin”,"midodrine/octreotide","noradr
      enaline"))

group$t.tx <-
  ifelse(!is.na(group$t.terl),group$t.terl,
    ifelse(!is.na(group$t.mido),group$t.mido,group$t.nadr))

group <- merge(group,
  study[,c(“study.id","design","N","year","author")])

group$study.group <- 1:nrow(group)

row.names(group) <- NULL

# Mortality data

mortality <- list(

data.frame(
group.id=1,
study.id=7496,
t=6,
died=1,
count=3),

data.frame(
group.id=1,
study.id=7496,
t=9,
died=1,
count=1),

data.frame(
group.id=1,
study.id=7496,
t=10,
died=1,
count=1),

data.frame(
```

```
group.id=1,
study.id=7496,
t=11,
died=1,
count=1),

data.frame(
group.id=1,
study.id=7496,
t=12,
died=1,
count=1),

data.frame(
group.id=1,
study.id=7496,
t=13,
died=1,
count=2),

data.frame(
group.id=1,
study.id=7496,
t=c(13,17,19,21,25,30),
died=rep(0,6),
count=c(3,1,1,1,1,4)),

data.frame(
group.id=2,
study.id=7496,
t=c(5,6,7,9,10,12,15,21,30),
died=c(rep(1,6),0,0,0),
count=c(1,3,2,1,1,1,2,2,7)),

data.frame(
group.id=1,
study.id=8454,
t=4,
died=1,
count=2),

data.frame(
group.id=1,
study.id=8454,
t=4,
died=0,
count=1),

data.frame(
group.id=1,
study.id=8454,
t=6,
died=1,
count=4),

data.frame(
group.id=1,
study.id=8454,
t=7,
died=1,
count=4),

data.frame(
group.id=1,
study.id=8454,
t=8,
died=1,
count=1),

data.frame(
group.id=1,
study.id=8454,
t=11,
died=1,
count=2),

data.frame(
group.id=1,
study.id=8454,
t=17,
died=1,
count=1),

data.frame(
group.id=1,
study.id=8454,
t=18,
died=1,
count=1),

data.frame(
group.id=1,
study.id=8454,
t=19,
died=0,
count=7),

data.frame(
group.id=2,
study.id=8454,
t=c(3,5,6,7,9,10,15,17,18,19),
died=c(rep(1,9),0),
count=c(1,2,2,3,1,1,2,1,2,8)),

data.frame(
group.id=1,
study.id=7541,
t=3,
died=1,
count=1),

data.frame(
group.id=1,
study.id=7541,
t=5,
died=1,
count=1),

data.frame(
group.id=1,
study.id=7541,
t=9,
died=1,
count=1),

data.frame(
group.id=1,
study.id=7541,
t=10,
died=1,
count=2),

data.frame(
group.id=1,
study.id=7541,
t=13,
died=1,
count=1),

data.frame(
```

```
group.id=1,
study.id=7541,
t=14,
died=1,
count=1),

data.frame(
group.id=1,
study.id=7541,
t=15,
died=1,
count=1),

data.frame(
group.id=1,
study.id=7541,
t=18,
died=1,
count=1),

data.frame(
group.id=1,
study.id=7541,
t=30,
died=1,
count=1),

data.frame(
group.id=1,
study.id=7541,
t=33,
died=1,
count=2),

data.frame(
group.id=1,
study.id=7541,
t=34,
died=1,
count=1),

data.frame(
group.id=1,
study.id=7541,
t=37,
died=1,
count=2),

data.frame(
group.id=1,
study.id=7541,
t=45,
died=0,
count=1),

data.frame(
group.id=1,
study.id=7541,
t=29,
died=0,
count=1),

data.frame(
group.id=1,
study.id=7541,
t=370,
died=0,
count=1),

data.frame(
```

```
group.id=1,
study.id=7462,
t=7,
died=1,
count=4),

data.frame(
group.id=1,
study.id=7462,
t=14,
died=1,
count=1),

data.frame(
group.id=1,
study.id=7462,
t=14,
died=0,
count=3),

data.frame(
group.id=1,
study.id=7462,
t=21,
died=1,
count=6),

data.frame(
group.id=1,
study.id=7462,
t=21,
died=0,
count=5),

data.frame(
group.id=1,
study.id=7462,
t=28,
died=1,
count=3),

data.frame(
group.id=1,
study.id=7462,
t=28,
died=0,
count=2),

data.frame(
group.id=1,
study.id=7462,
t=35,
died=1,
count=3),

data.frame(
group.id=1,
study.id=7462,
t=35,
died=0,
count=4),

data.frame(
group.id=1,
study.id=7462,
t=42,
died=1,
count=4),

data.frame(
```

```
group.id=1,
study.id=7462,
t=48,
died=0,
count=1),

data.frame(
group.id=1,
study.id=7462,
t=70,
died=1,
count=1),

data.frame(
group.id=1,
study.id=7462,
t=77,
died=0,
count=2),

data.frame(
group.id=1,
study.id=7462,
t=91,
died=0,
count=10),

data.frame(
group.id=1,
study.id=6602,
t=c(2,3,4,5,6,8,9,14,15,17,18,19,
   22,23,26,27,34,38,44,45,98,104,114,
   154,158,163,170,178:192,205,208,
   211,272,289),
died=c(rep(1,23),rep(0,24)),
count=c(5,2,2,1,2,2,rep(1,5),2,rep(1,11+24))),

data.frame(
group.id=1,
study.id=6739,
t=c(15,15-1,30,30-1,60,60-1,90,90-1,180,180-1,180+1),
died=c(1,0,1,0,1,0,1,0,1,0,0),
count=c(3,3,3,2,1,1,2,3,1,3,4)),

data.frame(
group.id=1,
study.id=7504,
t=c(2,4,6,8,9,10,11,12,13,15,16,18,20,
   22,24,25,27,29,30,31,32,34,39,40,42,
   43,62,68,83,86,98,113,
   24,25,27,33,42,49,52,60,63,76,103,
   118,180,360),
died=c(rep(1,32),rep(0,14)),
count=c(3,6,1,3,5,4,3,7,5,5,5,2,5,2,1,2,
   rep(1,16+13),11)),

data.frame(
group.id=1,
study.id=7505,
t=c(15,29,31,34,34,36,37,42,45,95,111),
died=c(rep(1,4),0,0,rep(1,4),0),
count=c(rep(1,7),2,rep(1,3))),

data.frame(
group.id=1,
study.id=7380,
t=c(14,15*30,10*30,30,60,360,6,12,4,8,12,16,20),
died=c(0,1,0,1,1,0,rep(1,7)),
count=rep(1,13)),

data.frame(
group.id=1,
study.id=8255,
t=c(2,23,27,34,36,40,84),
died=c(rep(1,6),0),
count=c(rep(1,5),2,1)),

data.frame(
group.id=1,
study.id=7497,
t=c(1,3,10,20,51),
died=c(1,0,0,0,0),
count=1),

data.frame(
group.id=2,
study.id=7497,
t=c(16,80,10,20),
died=c(1,1,0,0),
count=1),

data.frame(
group.id=1,
study.id=7543,
t=c(44,472,75,80,29),
died=c(0,0,1,0,1),
count=1),

data.frame(
group.id=1,
study.id=7518,
t=c(16,14,28,30,90,90,14,30,45,60),
died=c(0,rep(0,2),rep(1,7)),
count=c(5,rep(1,9))))

mortality <- do.call(rbind,mortality)

mortality <- as.data.frame(lapply(mortality,
  function(x) rep(x,mortality$count)))

mortality <- merge(mortality,group[,
  c(“group.id”,"study.id","study.group","n","dose.alb.tx",
    “t.alb.tx”,"dose.alb.cum","vaso.z","drug","t.tx","age",
    “scr.0”,”bilirubin","salb","map","design","N","year",
    “author”)])

# Predictor data

predictor <- list(

data.frame(
study.id=7503,
var="age",
responder.n=13,
responder=62,
responder.sd=10,
nonresponder.n=5,
nonresponder=57,
nonresponder.sd=7,
signif=FALSE),

data.frame(
study.id=7503,
var="scr",
responder.n=13,
responder=scrmol2mg(298),
responder.sd=scrmol2mg(124),
nonresponder.n=5,
nonresponder=scrmol2mg(257),
nonresponder.sd=scrmol2mg(78),
```

```
signif=FALSE),

data.frame(
study.id=7503,
var="bilirubin",
responder.n=13,
responder=bilimol2mg(155),
responder.sd=bilimol2mg(176),
nonresponder.n=5,
nonresponder=bilimol2mg(307),
nonresponder.sd=bilimol2mg(182),
signif=FALSE),

data.frame(
study.id=7503,
var="salb",
responder.n=13,
responder=2.6,
responder.sd=0.8,
nonresponder.n=5,
nonresponder=2.7,
nonresponder.sd=0.5,
signif=FALSE),

data.frame(
study.id=7503,
var="map",
responder.n=13,
responder=bp2map(124,64),
responder.sd=bp2map(16,14),
nonresponder.n=5,
nonresponder=bp2map(119,55),
nonresponder.sd=bp2map(13,13),
signif=FALSE),

data.frame(
study.id=7504,
var="age",
responder.n=53,
responder=53,
responder.sd=10,
nonresponder.n=38,
nonresponder=60,
nonresponder.sd=10,
signif=TRUE),

data.frame(
study.id=7504,
var="scr",
responder.n=53,
responder=scrmol2mg(263),
responder.sd=scrmol2mg(100),
nonresponder.n=38,
nonresponder=scrmol2mg(245),
nonresponder.sd=scrmol2mg(80),
signif=FALSE),

data.frame(
study.id=7518,
var="age",
responder.n=10,
responder=55.9,
responder.sd=2.3*sqrt(10),
nonresponder.n=4,
nonresponder=52.7,
nonresponder.sd=5.0*sqrt(4),
signif=FALSE),

data.frame(
study.id=7518,
var="scr",
responder.n=10,
responder=scrmol2mg(233),
responder.sd=scrmol2mg(29*sqrt(10)),
nonresponder.n=4,
nonresponder=scrmol2mg(345),
nonresponder.sd=scrmol2mg(83*sqrt(4)),
signif=FALSE),

data.frame(
study.id=7518,
var="bilirubin",
responder.n=10,
responder=bilimol2mg(45),
responder.sd=bilimol2mg(8*sqrt(10)),
nonresponder.n=4,
nonresponder=bilimol2mg(71),
nonresponder.sd=bilimol2mg(16*sqrt(4)),
signif=FALSE),

data.frame(
study.id=7518,
var="salb",
responder.n=10,
responder=3.2,
responder.sd=3*sqrt(10),
nonresponder.n=4,
nonresponder=3.3,
nonresponder.sd=5*sqrt(4),
signif=FALSE),

data.frame(
study.id=7518,
var="map",
responder.n=10,
responder=81,
responder.sd=5*sqrt(10),
nonresponder.n=4,
nonresponder=79,
nonresponder.sd=4*sqrt(4),
signif=FALSE),

data.frame(
study.id=7496,
var="age",
responder.n=20,
responder=47.0,
responder.sd=12.0,
nonresponder.n=20,
nonresponder=49.0,
nonresponder.sd=11.4,
signif=FALSE),

data.frame(
study.id=7496,
var="scr",
responder.n=20,
responder=3.0,
responder.sd=1.0,
nonresponder.n=20,
nonresponder=3.3,
nonresponder.sd=0.9,
signif=FALSE),

data.frame(
study.id=7496,
var="bilirubin",
responder.n=20,
responder=6.2,
responder.sd=(36.0-0.8)/3.5,
```

```
nonresponder.n=20,
nonresponder=6.9,
nonresponder.sd=(40.0-0.7)/3.5,
signif=FALSE),

data.frame(
study.id=7496,
var="salb",
responder.n=20,
responder=2.5,
responder.sd=0.5,
nonresponder.n=20,
nonresponder=2.6,
nonresponder.sd=0.5,
signif=FALSE),

data.frame(
study.id=7496,
var="map",
responder.n=20,
responder=83.0,
responder.sd=8.5,
nonresponder.n=20,
nonresponder=76.7,
nonresponder.sd=8.3,
signif=TRUE),

data.frame(
study.id=7380,
var="age",
responder.n=8,
responder=51.5,
responder.sd=5.3*sqrt(8),
nonresponder.n=5,
nonresponder=58.6,
nonresponder.sd=6.9*sqrt(5),
signif=FALSE),

data.frame(
study.id=7380,
var="scr",
responder.n=8,
responder=3.0,
responder.sd=1.7*sqrt(8),
nonresponder.n=5,
nonresponder=3.9,
nonresponder.sd=1.5*sqrt(5),
signif=FALSE),

data.frame(
study.id=7380,
var="map",
responder.n=8,
responder=70.1,
responder.sd=9.1*sqrt(8),
nonresponder.n=5,
nonresponder=68.8,
nonresponder.sd=6.5*sqrt(5),
signif=FALSE),

data.frame(
study.id=8454,
var="age",
responder.n=19,
responder=46.0,
responder.sd=10.1,
nonresponder.n=27,
nonresponder=52.6,
nonresponder.sd=11.9,
signif=FALSE),

data.frame(
study.id=8454,
var="scr",
responder.n=19,
responder=3.08,
responder.sd=0.6,
nonresponder.n=27,
nonresponder=3.27,
nonresponder.sd=0.7,
signif=FALSE),

data.frame(
study.id=8454,
var="bilirubin",
responder.n=19,
responder=4.8,
responder.sd=6.4,
nonresponder.n=27,
nonresponder=4.0,
nonresponder.sd=2.2,
signif=FALSE),

data.frame(
study.id=8454,
var="salb",
responder.n=19,
responder=2.8,
responder.sd=0.2,
nonresponder.n=27,
nonresponder=2.7,
nonresponder.sd=0.3,
signif=TRUE),

data.frame(
study.id=8454,
var="map",
responder.n=19,
responder=69.3,
responder.sd=9.3,
nonresponder.n=27,
nonresponder=61.9,
nonresponder.sd=11.2,
signif=TRUE),

data.frame(
study.id=7635,
var="map",
responder.n=19,
responder=72.8,
responder.sd=11.6,
nonresponder.n=37,
nonresponder=76.9,
nonresponder.sd=11.3,
signif=FALSE),

data.frame(
study.id=7488,
var="age",
responder.n=18,
responder=58,
responder.sd=9,
nonresponder.n=21,
nonresponder=55,
nonresponder.sd=10,
signif=FALSE),

data.frame(
study.id=7488,
var="scr",
responder.n=18,
```

```
responder=3.5,
responder.sd=1.4,
nonresponder.n=21,
nonresponder=3.9,
nonresponder.sd=1.4,
signif=FALSE),

data.frame(
study.id=7488,
var="bilirubin",
responder.n=18,
responder=6,
responder.sd=7,
nonresponder.n=21,
nonresponder=24,
nonresponder.sd=20,
signif=TRUE),

data.frame(
study.id=7488,
var="salb",
responder.n=18,
responder=3.0,
responder.sd=0.6,
nonresponder.n=21,
nonresponder=2.8,
nonresponder.sd=0.7,
signif=FALSE),

data.frame(
study.id=7488,
var="map",
responder.n=18,
responder=76,
responder.sd=13,
nonresponder.n=21,
nonresponder=79,
nonresponder.sd=9,
signif=FALSE)

)

predictor <- do.call(rbind,predictor)
```
